# Bio-fortification potential of global wild annual lentil core collection

**DOI:** 10.1371/journal.pone.0191122

**Published:** 2018-01-18

**Authors:** Sandeep Kumar, Anil Kumar Choudhary, Kuldeep Singh Rana, Ashutosh Sarker, Mohar Singh

**Affiliations:** 1 ICAR-National Bureau of Plant Genetic Resources, Pusa, New Delhi, India; 2 Department of Agronomy, ICAR- Indian Agricultural Research Institute, New Delhi, India; 3 International Centre for Agricultural Research in Dry Areas, South Asia and China Regional Programme (SACRP), DPS Marg Pusa Campus, New Delhi, India; 4 ICAR-National Bureau of Plant Genetic Resources Regional Station, Shimla, India; National Institute for Plant Genome Research, INDIA

## Abstract

Lentil, generally known as poor man’s’ meat due to its high protein value is also a good source of dietary fiber, antioxidants and vitamins along with fast cooking characteristics. It could be used globally as a staple food crop to eradicate hidden hunger, if this nutritionally rich crop is further enriched with essential minerals. This requires identification of essential mineral rich germplasm. So, in the present study, a core set of 96 wild accessions extracted from 405 global wild annual collections comprising different species was analyzed to determine its bio-fortification potential. Impressive variation (mg/100 g) was observed for different minerals including Na (30–318), K (138.29–1578), P (37.50–593.75), Ca (4.74–188.75), Mg (15–159), Fe (2.82–14.12), Zn (1.29–12.62), Cu (0.5–7.12), Mn (1.22–9.99), Mo (1.02–11.89), Ni (0.16–3.49), Pb (0.01–0.58), Cd (0–0.03), Co (0–0.63) and As (0–0.02). Hierarchical clustering revealed high intra- and inter-specific variability. Further, correlation study showed positive significant association among minerals and between minerals including agro-morphological traits. Accessions representation from Turkey and Syria had maximum variability for different minerals. Diversity analysis exhibited wide geographical variations across gene-pool in core set. Potential use of the identified trait-specific genetic resources could be initial genetic material, for genetic base broadening and biofortification of cultivated lentil.

## Introduction

On earth, at least half the human population do not get sufficient daily amounts of essential minerals, a condition leading to ‘hidden hunger’ either due to their low concentrations in commonly eaten staple food crops or their reduced bioavailability [1]. Among minerals, deficiencies specifically of iron exists within nearly 3.7 billion people worldwide comprising about 60% of the whole population [2], whereas zinc affecting about one-third of the world population [3], are the most common. Adequate amounts of copper, manganese, molybdenum, cobalt, nickel, sodium and potassium are also required in body as these act as components of various enzymes or vitamins [4] or involved in other metabolic activities [5]. Atleast one physiological function is known for each of the nine trace elements namely iron, zinc, copper, manganese, molybdenum, chromium, fluoride, iodine and selenium in human metabolism and these are ranked as essential micronutrients and should be consumed regularly in food or as supplements [6, 7]. So, there is an urgent need to identify or develop value added material for the essential micronutrients through fortification/bio-fortification particularly in crops that can be used as staple food, to eradicate the problem of hidden hunger.

Lentil (*Lens culinaris* ssp. *culinaris*) is the fourth most important pulse crop after bean (*Phaseolus vulgaris* L.), pea (*Pisum sativum* L.), and chickpea (*Cicer arietinum* L.). It is grown widely throughout the Indian sub-continent, the Middle East, Northern and East Africa, Southern Europe, North and South America, Australia and western Asia [8]. The major lentil producing countries in the world are Canada, India, Turkey, Australia, USA, Nepal, China, and Ethiopia. The world annual production and productivity of lentil is estimated up to 4.95 million tons and 1260 kg/ha, respectively, from cultivated area of around 4.34 million ha [9]. Lentil is known as poor man’s’ meat, due to its high protein value and its fast cooking characteristics. It is also a good source of dietary fiber, minerals and vitamins, including other functional compounds particularly antioxidants [10]. Lentil being a nutritious crop, well adapted to dry land agro-ecologies with poor soil fertility and crop management situations, provides an alternative to be used as staple crop to eradicate the global hidden hunger [11].

Crop wild relatives (CWRs) are reservoir of important traits particularly resistance to biotic and abiotic stresses and nutritional parameters. In our previous study, we have developed a wild lentil core set from global collections and identified some promising accessions for yield contributing traits including disease resistance [12], which are currently being used for genetic base broadening of the cultivated varieties. On the other hand, legume wild species have also been reported as a rich source of various minerals [13]. But scarce information is available on elemental composition of wild lentil species. So in this study, wild lentil core set developed from global collections representing wide range of genetic diversity was analyzed to determine variations in mineral composition with an aim to find out its bio-fortification potential.

## Materials and methods

### Materials

A total of 96 wild lentil accessions including species *L*. *culinaris* ssp. *orientalis* (24 accs.), *L*. *culinaris* ssp. *odemensis* (16 accs.), *L*. *culinaris* ssp. *tomentosus* (8 accs.), *L*. *ervoides* (24 accs.), *L*. *nigricans* (17 accs.), *L*. *lamottei* (5 accs.) and cultivated species *L*. *culinaris* ssp. *culinaris* (2 accs.) were grown in plastic pots (size 24” x 24”filled with 10 kg of sandy loam soil in naturally lit net house at Germplasm Evaluation Division, Indian Council of Agricultural Research-National Bureau of Plant Genetic Resources (ICAR-NBPGR), New Delhi during 2013–14 and 2014–15. The details of accessions and their geographical origin are listed in Table 1. Seeds were collected at harvest stage and analyzed for various mineral elements. All the chemicals including standards used in the present study were of high purity.

**Table 1:** List of wild lentil core set accessions along with their country origin.

S. No.	Species/accessions	Origin	S. No.	Species/Accessions	Origin
	***L*. *culinaris* ssp. *orientalis***			***L*. *lamottei***	
1	ILWL 7	Turkey	50	ILWL 14	France
2	ILWL 8	Turkey	51	ILWL 15	France
3	ILWL 75	Israel	52	ILWL 29	Spain
4	ILWL 89	Turkey	53	ILWL 429	Spain
5	ILWL 95	Turkey	54	EC718692	France
6	ILWL 96	Turkey		***L*. *culinaris* ssp. o*demensis***	
7	ILWL 101	Turkey	55	ILWL 20	Palestine
8	ILWL 117	Syria	56	ILWL 35	Turkey
9	ILWL 124	Syria	57	ILWL 56	Palestine
10	ILWL 181	Syria	58	ILWL 165	Syria
11	ILWL 227	Syria	59	ILWL 166	Syria
12	ILWL 230	Syria	60	ILWL 167	Syria
13	ILWL 243	Syria	61	ILWL 196	Not known
14	ILWL 246	Syria	62	ILWL 235	Syria
15	ILWL 278	Turkey	63	ILWL 320	Turkey
16	ILWL 330	Syria	64	ILWL 357	Syria
17	ILWL 343	Syria	65	ILWL 361	Syria
18	ILWL 344	Syria	66	ILWL 409	Syria
19	ILWL 349	Syria	67	ILWL 436	Turkey
20	ILWL 359	Syria	68	ILWL 438	Turkey
21	ILWL 384	Tajikistan	69	EC718311	Israel
22	ILWL 443	Turkey	70	EC718694	Syria
23	ILWL 476	Turkey		***L*. *ervoides***	
24	ILWL 480	Syria	71	ILWL 30	Spain
	***L*. *nigricans***		72	ILWL 43	Croatia
25	ILWL 9	Syria	73	ILWL 50	Croatia
26	ILWL 14	Syria	74	ILWL 51	Montenegro
27	ILWL 15	France	75	ILWL 58	Turkey
28	ILWL16	Alpes-Cote d'Azur	76	ILWL 60	Turkey
29	ILWL 18	France	77	ILWL 61	Turkey
30	ILWL 19	Spain	78	ILWL 63	Turkey
31	ILWL 22	Italy	79	ILWL 65	Turkey
32	ILWL 31	Spain	80	ILWL 66	Turkey
33	ILWL 34	Ukraine	81	ILWL 92	Turkey
34	ILWL 37	Turkey	82	ILWL 234	Syria
35	ILWL 38	Turkey	83	ILWL 269	Turkey
36	ILWL 191	Croatia	84	ILWL 276	Turkey
37	ILWL 460	Turkey	85	ILWL 292	Turkey
38	EC718266	Italy	86	ILWL 321	Turkey
39	EC718270	Croatia	87	ILWL 398	Lebanon
40	EC718273	Spain	88	ILWL 401	Lebanon
41	EC718275	Turkey	89	ILWL 408	Syria
	***L*. *culinaris* ssp. *tomentosus***		90	ILWL 414	Syria
42	ILWL 90	Turkey	91	ILWL 418	Syria
43	ILWL 195	Syria	92	ILWL 441	Turkey
44	ILWL 198	Syria	93	ILWL 442	Turkey
45	ILWL 199	Syria	94	EC718439	Israel
46	ILWL 305	Turkey		***L*. *culinaris* ssp. *culinaris***	
47	ILWL 308	Turkey	95	ILL 8006	Syria
48	ILWL 480	Syria	96	ILL 10829	Syria
49	EC718673	Syria			

### Sample preparation

Dried seeds of different accessions were grinded using pastle and mortar. To 500 mg of powdered seed samples, 10 ml of concentrated nitric acid (70% HNO_3_) was added and kept for overnight. Samples were then heated to 90°C for 16 h in digestion chamber as suggested by Zarcinas et al. [14]. The digested samples were then filtered and made up to 100 ml with Milli-Q water.

### Mineral analysis

Sodium (Na) and potassium (K) were determined using flame photometer 128 (Systronics), phosphorus (P) by using spectrophotometer DR5000 (HACH). Calcium (Ca) and magnesium (Mg) were determined by using Atomic Absorption spectrophotometer (AnalytiKjena ZEEnit 700, Germany). Other mineral elements namely, iron, copper, zinc, manganese, molybdenum, nickel, lead, cadmium, cobalt and arsenic were determined using ICP-Mass Spectrophotometer (NexIon^®^ 300X Series, Perkin-Elmer). All the standards of different mineral elements used in the study were of analytical grade. All the observations were recorded in two replications.

### Statistical and diversity analysis

Descriptive statistics analysis was done using Windows 2007 Microsoft Excel Data Analysis Pack [15]. Phenotypic coefficient of variation (PCV) for different elements were calculated as PCV = √V_P_/ mean x 100as per Burton [16]. Genetic diversity among accessions based on elemental composition data was determined from the Euclidean distances. Hierarchical clustering and correlation analysis along with Principal Component Analysis (PCA) was carried out using SAS software [17].

## Results

### Mineral composition of core collection

The accessions comprising wild lentil core collection exhibited wide range of variability for different minerals including Na (30–318 mg/100 g), K (138.29–1578 mg/100 g), P (37.50–593.75 mg/100 g), Ca (4.74–188.75 mg/100 g), Mg (15–159 mg/100 g), Fe (2.82–14.12 mg/100 g), Zn (1.29–12.62 mg/100 g), Cu (0.5–7.12 mg/100 g), Mn (1.22–9.99 mg/100 g), Mo (1.02–11.89 mg/100 g), Ni (0.16–3.49 mg/100 g), Pb (0.01–0.58mg/100 g), Cd (0–0.03 mg/100 g), Co (0–0.63 mg/100 g) and As (0–0.02 mg/100 g). The species-specific mineral composition of lentil accessions is listed in Table 2. In general, the coefficient of variation (CV) was higher in magnitude for almost all elements. The mineral data obtained in the present study was compared with the literature values available in S1 Table. The data clearly indicated that the range of various minerals was higher in *Lens* core set accessions. The essential minerals important for bio-fortification purposes are further described in detail.

**Table 2:** Species-wise mineral composition of wild lentil core accessions.

Minerals (mg/100g)															
Species	Na	K	P	Ca	Mg	Fe	Zn	Cu	Mn	Mo	Ni	Pb	Cd	Co	As
***L*. *culinaris* ssp.*orientalis*(24)**															
MIN	92.00	138.29	37.5	18.63	46.00	4.48	1.29	0.5	1.22	1.62	0.21	0.01	0.00	0.00	0.00
MAX	316.00	1453.54	250	136.62	158.00	14.12	12.46	5.01	9.56	11.89	3.49	0.58	0.03	0.63	0.02
AVERAGE	196.08	1153.98	134.61	52.07	98.04	7.76	5.94	2.52	5.66	7.23	1.13	0.32	0.01	0.12	0.00
CV	37.91	25.47	37.78	55.52	37.91	36.57	45.39	50	42.57	27.8	84.07	34.37	0.00	116.66	0.00
SE	15.17	60.02	10.38	5.9	7.59	0.58	0.55	0.25	0.49	0.41	0.19	0.02	0.00	0.02	0.00
***L*. *nigricans* (17)**															
MIN	164.00	1022.50	72.50	10.47	82.00	6.91	4.96	1.21	2.16	2.05	0.3	0.36	0.01	0.01	0.00
MAX	312.00	1556.00	265.00	188.75	156.00	13.56	12.62	5.24	8.34	11.77	1.05	0.56	0.03	0.07	0.01
AVERAGE	234.82	1201.08	144.30	62.35	117.41	9.73	7.90	2.66	4.53	6.75	0.71	0.4	0.02	0.03	0.00
CV	19.36	12.51	37.8	65.93	19.35	20.87	32.11	36.84	49.44	39.55	26.76	10	0.00	33.33	0.00
SE	11.03	36.44	13.23	9.97	5.51	0.49	0.61	0.23	0.54	0.64	0.04	0.01	0.00	0.00	0.00
***L*. *culinaris*ssp. *tomentosus* (8)**															
MIN	196.00	1094.00	41.66	16.13	98.00	5.24	3.41	1.21	2.01	1.36	0.52	0.31	0.00	0.00	0.00
MAX	318.00	1578.00	170	83.97	159.00	10.18	9.11	6.2	9.28	11.27	1.8	0.56	0.02	0.26	0.02
AVERAGE	269.88	1345.50	120.2	44.42	134.94	7.14	5.45	2.18	4.51	7.11	1.05	0.37	0.01	0.07	0.00
CV	15.78	12.67	37.16	50.85	15.78	24.62	36.19	77.06	48.33	45.99	49.52	18.91	0.00	114.28	0.00
SE	15.06	60.29	15.79	7.98	7.53	0.62	0.70	1.59	0.77	1.15	0.18	0.02	0.00	0.03	0.00
***L*. *lamottei* (5)**															
MIN	196.00	1176.00	102.50	19.11	98.00	5.05	4.12	2.31	3.7	1.37	0.89	0.14	0.00	0.02	0.00
MAX	298.00	1542.00	205.00	72.74	149.00	13.95	12.60	4.67	8.83	11.11	3.08	0.52	0.02	0.10	0.02
AVERAGE	265.20	1289.20	134.00	32.19	132.60	10.74	9.56	3.4	5.66	6.85	2.06	0.32	0.01	0.05	0.01
CV	15.03	11.53	32.16	70.86	15.03	35.42	38.47	25.58	42.75	58.24	38.34	50.0	0.00	60.0	0.00
SE	17.82	66.47	19.27	10.2	8.91	1.70	1.64	0.39	1.08	1.78	0.35	0.07	0.00	0.01	0.00
***L*. *culinaris*ssp. *odemensis*(16)**															
MIN	158.00	1074	87.5	8.48	79.00	2.82	2.80	1.18	1.71	1.88	0.16	0.15	0.00	0.00	0.00
MAX	310.00	1538	205	37.34	155.00	14.12	12.41	7.03	9.76	11.85	2.91	0.46	0.02	0.16	0.02
AVERAGE	251.44	1318.62	133.65	17.93	125.72	9.97	7.34	2.8	4.33	5.94	0.76	0.26	0.01	0.04	0.00
CV	21.37	11.51	25.16	49.63	21.37	31.38	40.07	52.14	51.27	50.16	94.73	26.92	0.00	100	0.00
SE	13.44	37.95	8.4	2.22	6.72	0.78	0.73	0.36	0.55	0.74	0.18	0.01	0.00	0.01	0.00
***L*. *ervoides* (24)**															
MIN	72.50	560	172.5	6.98	36.25	4.58	2.50	1.27	3.02	1.02	0.17	0.07	0.00	0.01	0.00
MAX	316.67	1563.33	593.75	71.38	158.33	12.64	9.84	7.12	9.99	11.31	3.2	0.53	0.02	0.29	0.01
AVERAGE	204.47	1071.3	295.24	20.97	102.24	8.73	6.88	3.64	5.85	4.81	0.95	0.28	0.01	0.06	0.00
CV	31.38	18.42	38.7	70.33	31.39	23.59	28.93	58.24	38.11	57.17	80	32.14	0.00	100	0.00
SE	13.10	40.3	23.32	3.01	6.55	0.42	0.41	0.43	0.45	0.56	0.15	0.01	0.00	0.01	0.00
**L. culinaris ssp. culinaris (2)**															
MIN	30	1030	365	4.74	15.00	5.37	4.92	2.46	3.2	1.22	0.87	0.30	0.00	0.05	0.00
MAX	54	1088	392.5	5.62	27.00	6.07	5.16	2.57	3.93	1.93	1.08	0.31	0.00	0.06	0.00
AVERAGE	42	1059	378.75	5.18	21.00	5.72	5.04	2.52	3.57	1.57	0.98	0.30	0.00	0.05	0.00
CV	40.41	3.87	5.13	11.96	40.40	8.65	3.36	3.17	14.28	31.84	15.3	0.00	0.00	20.0	0.00
SE	12	29	13.75	0.44	6.00	0.35	0.12	0.05	0.36	0.35	0.1	0.00	0.00	0.00	0.00
***Across the species***															
MIN	30.00	138.29	37.50	4.74	15.00	2.82	1.29	0.50	1.22	1.02	0.16	0.01	0.00	0.00	0.00
MAX	318	1578	593.75	188.75	159.00	14.12	12.62	7.12	9.99	11.89	3.49	0.58	0.03	0.63	0.02
AVERAGE	220.80	1190.11	180.18	37.78	110.40	8.84	6.9	2.89	5.14	6.18	0.98	0.32	0.01	0.07	0.00
SE	7.02	22.93	10.41	3.12	3.51	0.27	0.23	0.16	0.23	0.29	0.07	0.01	0.00	0.00	0.00
CV	31.17	18.88	56.62	81.02	31.17	31.9	18.69	54.30	44.82	46.47	77.37	31.90	57.14	-	-

The maximum variability for Ca content was observed in *L*. *nigricans* with 10.47 mg/100g (EC718266) to 188.75 mg/100 g (ILWL9) whereas, minimum variability was observed in *L*. *culinaris* ssp. *culinaris* from 4.74mg/100 g (ILL8006) to 5.63 mg/100 g (ILL10829) followed by *L*. *culinaris* ssp. *odemensis* with 8.48 mg/100 g (ILWL357) to 37.34 mg/100 g (ILWL35). Accessions ILWL9 of *L*. *nigricans* from Syria and ILWL278 and ILWL343 of *L*. *culinaris* ssp. *orientalis* from Turkey and Syria, had Ca content >100 mg/100 g with 188.75, 136.63, and 113.37 mg/100 g, respectively. Species *L*. *ervoides* and *L*. *culinaris* ssp. *orientalis* showed substantial variability with 36.25 to 158.33 and 46 to 158 mg/100 g, respectively, for the Mg content. *L*. *culinaris* ssp. *tomentosus* accession ILWL90 from Turkey had highest Mg content (159 mg/100 g). Accessions ILWL401, ILWL7, ILWL117, ILWL37, ILWL480 and ILWL66 had Mg content of >155 mg/100 g.

In case of Fe, maximum variability of 2.82 (ILWL20) to 14.12 (EC718311) mg/100 g was observed in *L*. *culinaris* ssp. *odemensis* followed by *L*. *culinaris* ssp. *orientalis* with 4.48 (ILWL75) to 14.12 (ILWL243) mg/100 g. However, *L*. *culinaris* ssp. *tomentosus* showed limited variability for Fe content with 5.24 (EC718673) to 10.18 (ILWL305) mg/100 g. All other species also showed reasonable variability for Fe content (Table 2). Accessions namely ILWL243 (14.12 mg/100 g), EC718311 (14.12 mg/100 g), EC718692 (13.95 mg/100 g), ILWL357 (13.62 mg/100 g), EC718275 (13.56 mg/100 g) had higher Fe content. Other accessions with higher Fe content are listed in Table 3. In case of Zn, maximum variability was observed in *L*. *culinaris* ssp. *orientalis* followed by *L*. *culinaris* ssp. *odemensis* as evident from the Table 2. Accessions from *L*. *lamottei* and *L*. *nigricans* showed almost identical range for Zn with 4.12 to 12.60 and 4.96 to 12.62 mg/100 g, respectively. Similarly, *L*. *culinaris* ssp. *tomentosus* and *L*. *ervoides* also covered the same range with 3.41 to 9.11 and 2.5 to 9.84 mg/100 g, respectively. Among the accessions, EC718275 of *L*. *nigricans* belonging to Turkey showed highest value of 12.62 mg/100 g followed by EC718692 of *L*. *lamottei* from France with 12.60 mg/100 g of Zn content. In case of Cu content, maximum variability observed among accessions from *L*. *ervoides* and *L*. *culinaris* ssp. *odemensis* with 1.27 (ILWL66) to 7.13 (ILWL43), and 1.18 (ILWL361) to 7.03 (ILWL56) mg/100 g, respectively. Accessions ILWL 43 (7.13 mg/ 100 g) from *L*. *ervoides* and ILWL 56 (7.03 mg/100 g) from *odemensis* had higher Cu content. The species *L*. *lamottei* had a limited range of variation from 2.31 (ILWL429) to 4.68 (ILWL29) followed by *L*. *culinaris* ssp. *culinaris* with 2.46 (ILL8006) to 2.58 (ILL10829) mg/100 g. The maximum variability for Mn content was observed among accessions from *L*. *Culinaris* ssp. *orientalis* with 1.22 (ILWL359) to 9.57 (ILWL330) and *L*. *culinaris* ssp. *odemensis* with 1.71 (ILWL320) to 9.77 (ILWL56), mg/100 g. However, ILWL 269 (9.99 mg/100 g) of *L*. *ervoides* from Turkey, ILWL 56 (9.77 mg/100 g) of *L*. *culinaris* ssp. *odemensis* from Palestinian and ILWL 330 (9.57 mg/100 g) of *L*. *culinaris* ssp. *orientalis* from Syria were found with higher Mn content in comparison to other accessions. Highest Mo content was found in *L*. *culinaris* ssp. *orientalis* accession ILWL476 (11.89 mg/100 g) from Turkey followed by *L*. *culinaris* ssp. *odemensis* accession EC718311 (11.85 mg/100 g) from Israel.

**Table 3:** Promising accessions identified for some important minerals for their use in lentil improvement programme.

Species	Ca*	Mg*	Fe*	Zn*	Cu*	Mn*	Mo*
***L*. *culinaris* ssp. orientalis**	ILWL 278 (136.63)	ILWL 7 (158.00)	ILWL 243 (14.12)	ILWL 117 (12.46)	ILWL 476 (5.02)	ILWL 330 (9.57)	ILWL 476 (11.90)
	ILWL343 (113.37)	ILWL117 (157.00)	ILWL117 (12.62)	ILWL 480(11.03)	ILWL 443 (4.81)	ILWL 443 (9.20)	ILWL 349 (10.74)
	ILWL124 (99.43)	ILWL480 (155.00		ILWL349 (10.61)			
		ILWL95 (146.00)		ILWL230 (9.14)			
		ILWL343 (145.65)					
***L*. *nigricans***	ILWL 9 (188.75)	ILWL 37(156.00)	EC718275(13.56)	EC718275(12.62)	EC718273 (5.24)	ILWL 34 (8.35)	ILWL 16 (11.77)
	ILWL15 (99.0)	ILWL 9 (148.75)	ILWL 15 (12.97)	ILWL15 (12.36)		ILWL 16 (8.33)	ILWL 34 (11.77)
	ILWL14 (95.62)	ILWL31 (146.25)	ILWL22 (12.16)				
***L*. *culinaris* ssp. *tomentosus***	ILWL 305 (83.97)	ILWL 90 (159.00)	ILWL 305 (10.18)	ILWL 305 (9.11)	ILWL 480 (6.20)	ILWL 480 (9.28)	ILWL 308 (11.27)
	ILWL480 (64.83)	ILWL480 (155.50)					ILWL 480 (11.23)
		ILWL199 (148.00)					
		EC718673 (147.00)					
***L*. *lamottei***	ILWL 14 (72.74)	ILWL 14 (149.00)	EC718692 (13.95)	EC718692 (12.60)	ILWL 29 (4.68)	ILWL 15 (8.84)	ILWL 15 (11.12)
		ILWL 15 (972.30)	ILWL 15 (13.32)	ILWL 15 (12.48)		ILWL 29 (7.70)	
***L*. *culinaris* ssp. o*demensis***	ILWL 35 (37.34)	EC718311(155.00)	EC718311(14.12)	EC718694 (12.41)	ILWL 56 (7.03)	ILWL 56 (9.77)	EC718311 (11.85)
		Ilwl35 (152.50)	ILWL357 (13.62)	ILWL357 (12.37)			
		ILWL56 (151.00)	ILWL409 (13.32)				
		ILWL166 (150.00)					
***L*. *ervoides***	ILWL 51 (71.38)	ILWL 401(158.33)	ILWL58 (12.64)	ILWL 441 (9.84)	ILWL 43 (7.13)	ILWL 269 (9.99)	ILWL 269 (11.31)
	ILWL401 (51.73)	ILWL66 (156.00)	ILWL441 (11.90)	ILWL401 (9.24)	ILWL92 (6.93)		
		ILWL58 (153.00)	ILWL30 (11.66)	ILWL276 (9.23)	ILWL441 (6.79)		
			ILWL 401 (11.58)		ILWL276 (6.57)		
					ILWL50 (6.32)		

*Data in parenthesis is in mg/100 g.

Some accessions namely ILWL15 of *L*. *lamottei* found promising for Mg, Fe, Zn, Mn; Mo and ILWL480 of *L*. *culinaris* ssp. *odemensis* for Ca, Mg, Cu, Mn, Mo; and ILWL401 of *L*. *ervoides* for Ca, Mg, Fe and Zn. Promising accessions from different species rich in various minerals identified are listed in Table 3.

### Diversity analysis

**Fig 1:**
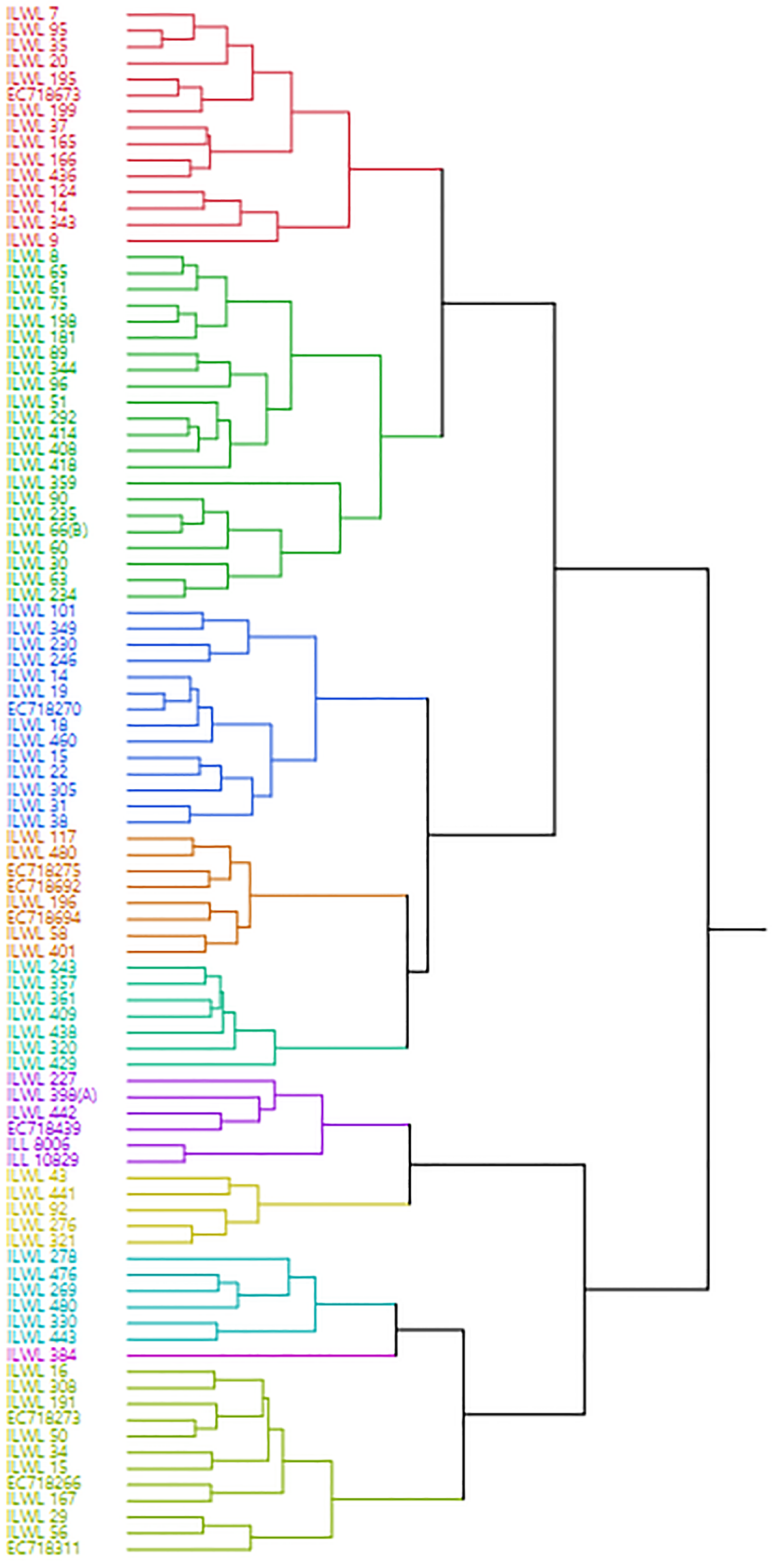
Hierarchical clustering of 96 wild lentil accessions comprising core set based on mineral composition data.

Diversity analysis was done using data of all the 15 minerals analysed in the study. Euclidean distances ranged from 0.845 to 14.291 as given in S2 Table. The maximum distance of 14.291 was observed between accessions ILWL7 from Turkey and ILWL227 *from Syria* of *L*. *culinaris* ssp. *orientalis*, which was followed by *L*. *culinaris* ssp. *orientalis* accessions ILWL227 from Syria and ILWL278 from Turkey with a value of 11.248, ILWL7and ILWL101, both from Turkey with a distance value of 10.518, and ILWL278 of *L*. *culinaris* ssp. *orientalis* from Turkey and ILWL16 of *L*. *nigricans* from Alpes-Cote d’Azur with 8.268. The minimum distance or maximum similarity was observed between ILWL95 of *L*. *culinaris* ssp. *orientalis* and ILWL35 of *L*. *culinaris* ssp. *odemensis*, both from Turkey with a Euclidean distance of 0.845 followed by *L*. *nigricans* accessions ILWL19 from Spain and EC718270 from Croatia with Euclidean distance of 0.90, and ILWL75 of *L*. *culinaris* ssp. *orientalis* from Israel and ILWL198 of *L*. *culinaris* ssp. *tomentosus* from Syria with a distance of 1.238. The hierarchical clustering of accessions resulted into9different clusters A-J as shown in different colors in Fig 1. All the clusters occupied by the accessions of different species, but in some clusters majority of the accessions were observed species specific. Cluster C was mainly occupied by *L*. *nigricans*, E by *L*. *culinaris* ssp. *odemensis*, F by *L*. *ervoides* and *L*. *culinaris* ssp. *culinaris*, G by *L*. *ervoides*, H by *L*. *culinaris* ssp. *orientalis*. ILWL384 forms a separate cluster I. The clustering pattern indicates that grouping of accessions was not according to their geographical origins as shown in S2 Table.

### Principal Component Analysis

**Fig 2:**
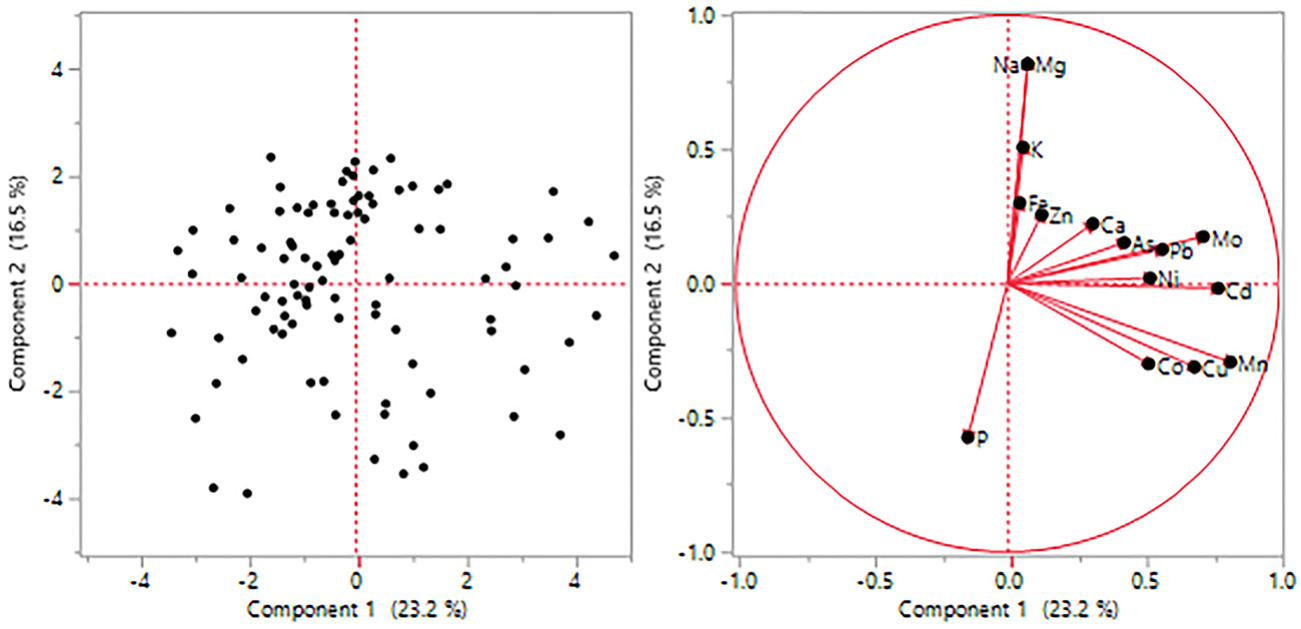
PCA analysis—(2a) 2-D graph of first two principal components and (2b) various mineral elements contributing to the variability.

Principal Component Analysis was used to assess the patterns of variation by considering all variables simultaneously. First four components have eigen value >1.0 which together explained 65.09 per cent of total variability Table 4. PC 1 accounted for 23.2% of the total variation, and, Mn, Cd, Cu and Mo had the highest positive coefficients means that they were the most variable traits. Similarly, PC2 explained 16.5% of the total variation, and Na and Mg had the highest positive coefficients. PC3 accounted for 15.4% of the total variation and Fe and Zn had the highest positive coefficients. The traits contributing to diversity are listed in Table 4. Furthermore, the two dimensional scattering of core set accessions according to PCA and various traits contributing to the variation are shown in Fig 2a and 2b, respectively.

**Table 4:** Eigenvectors, eigen values, individual and cumulative percentages of variation explained by the first five principal components (PC) of 96 wild lentil accessions.

Minerals	Prin1	Prin2	Prin3	Prin4
**Na**	0.039	0.519	-0.214	0.314
**K**	0.031	0.323	0.235	-0.034
**P**	-0.078	-0.364	0.173	0.345
**Ca**	0.168	0.142	-0.296	-0.366
**Mg**	0.039	0.519	-0.214	0.314
**Fe**	0.024	0.191	0.530	0.075
**Zn**	0.067	0.163	0.532	0.078
**Cu**	0.369	-0.198	0.148	0.295
**Mn**	0.440	-0.186	-0.041	0.232
**Mo**	0.386	0.111	0.043	-0.239
**Ni**	0.281	0.013	-0.092	0.297
**Pb**	0.305	0.081	0.182	-0.373
**Cd**	0.416	-0.011	0.014	-0.256
**Co**	0.278	-0.190	-0.307	0.148
**As**	0.230	0.097	0.094	0.151
**Eigen value**	3.47	2.47	2.31	1.50
**Percent**	23.15	16.47	15.44	10.03
**Cumulative Percent**	23.15	39.62	55.06	65.09

### Association between mineral elements and with agro-morphological traits

The correlation coefficients among the different mineral elements and with agro-morphological traits are shown in S3 Table. Correlation analysis indicated numerous significant positive and negative correlations. As test power increased with large number of observations, giving significance to most of correlations, results only with with *r*-values greater than 0.4 are discussed here. Fe showed significant positive correlations with Zn (*r* = 0.793); Mg with Na (0.720); Cu with Mn (*r* = 0.753) and Cd (*r* = 0.480); Mn with Ni (*r* = 0.436), Cd (*r* = 0.511), Co (*r* = 0.576) and Mo (*r* = 0.441); Mo with Pb (*r* = 0.429), Cd (*r* = 0.615); Ni with Co (*r* = 0.429); and, Pb with Cd (*r* = 0.525).

## Discussion

### Elemental composition of core set accessions

The wild annual lentil core accessions used in the present study developed from the global wild lentil germplasm accessions introduced from twenty seven counties [12]. An idea was to study the core set accessions for detailed evaluation, which seems to be much useful from utilization point of view. Further, it makes easy to handle reduced number of accessions for detailed studies including biochemical, physiological and molecular analysis. Earlier also, core and mini core collections have been evaluated for nutritional traits [18, 19].

The accessions showed high variability for different minerals because core set includes accessions from five different wild species belonging to the diverse originswith different genetic backgrounds. Further, core set retains the genetic diversity of the whole population from which it is developed [20]. The partitioning of mineral elements is affected by grain morphology including grain size, embryo size, and thickness of the tissue layers. In wild and cultivated wheat, variability in grain for Fe and Zn contents have been found related to allelic variation at a chromosomal locus involved in remobilization of protein, Fe, Zn and Mn from senescing leaves to seeds [21]. Recently, Gupta et al. [22] developed markers for some iron metabolism related genes (*Ferritin-1*, *BHLH-1*, *IRT-1*) in lentil. Thus, the intra- as well as inter-specific variability observed for seed elemental composition in the present study also highlights the hidden role of tight genetic control besides influence of plant morphology. Thus, the data generated provides a valuable resource for mapping studies to identify genes or points of regulation for elemental variation.

In general, species *L*. *nigricans* and *L*. *culinaris* ssp. *orientalis* showed wide variability for Ca content, whereas *L*. *ervoides* and *L*. *culinaris* ssp. *orientalis*, for Mg content. Species *L*. *culinaris* ssp. *odemensis*, *L*. *culinaris* ssp. *orientalis* and *L*. *ervoides* showed remarkable variability for different minerals viz. Fe, Zn, Cu, Mn and Mo. This may be occurred due to diverse genetic makeup of these species or may be due to the sound expression of respective genes of particular species under given environmental conditions. However, *L*. *culinaris* ssp. *culinaris* had the minimum variability for some of the minerals as it is a cultivated species. Singh et al. [23] revealed significant effects for genotype, environment and genotype by environment interaction for both grain Fe and Zn concentration in 50 lentil accessions.

The results revealed that in general accessions originated from Syria and Turkey had the maximum variability for different essential minerals. The study can help us in revisiting of Syria and Turkey regions for more useful wild annual lentil species germplasm collections through explorations. Laghetti et al. [24] analyzed some important Italian lentil landraces for Na (10.85–23.00 mg/100 g), P (405.4–542.4 mg/100 g), K (916.8–1071.8 mg/100 g), and Ca (49.57–59.79 mg/ 100 g) contents. Zia-Ul Haq et al. [25] systematically analyzed four improved lentil cultivars viz., Masoor 85, Masoor 93, NIAB Masoor 2002 and NIAB Masoor 2006 and determined Fe (2.7–3.2mg/100 g), Cu (8.9–9.9mg/100 g), Zn (3.9–4.6mg/100 g), and Mn (1.4–4.3mg/100 g). The micronutrient contents in Ethiopian lentil samples have been reported in the range of 0.226 to 0.282 mg/100 g for Cu, 9.17 to 11.91 mg/100 g for Fe, 6.7 to 8.2 mg/100 g for Mn, and 8.62 to 10.03 mg/100 g for Zn [26]. Alghamdi et al. [27] assessed mineral diversity among 35 introduced domesticated lentil genotypes and reported pronounced variations with K from 674.4 to 1061.2 mg/100 g, P 286.9 to 546.7 mg/100 g, Mg 126.1 to 157.3 mg/100 g and Ca 64.9 to 84 mg/100 g, iron 6.57 to 8.57 mg/100 g, zinc from 2.63 to 4.51 mg/100 g, manganese 1.26 to 2.85 mg/100 g and copper 0.86 to 1.37 mg/100 g.

In the present study, Ni, Pb, Cd, Co and As were found almost within recommended range except two-three accessions (Table 2). The data suggests that wild Lens accessions are not good accumulators of these minerals. Leshe and Tessema [26] reported mineral composition of Ethiopian lentil samples in the range Ni (0.120 to 0.244 mg/100 g), Pb (0.142 to 0.176 mg/100 g), Cd (0.009 to 0.013 mg/100 g) and Co (0.285 to 0.360 mg/100 g).

The Comparison of mineral data of lentil wild species obtained in the present study with that of the literature values of cultivated material in S1 Table highlighted that wild species are rich source of various minerals. The variation reported from earlier studies could be due to diverse genetic constitution, species-specificity, origin and environmental differences. These untapped gene sources identified in the present study can be hybridized with cultivated lentil varieties for genetic improvement of nutritional traits [28]. Earlier also, value rich accessions have been identified for various minerals and other nutritional constituents viz. 10 accessions each for high iron and zinc in sorghum seed, 1 accession for iron and 2 accessions for zinc in pearl millet seed, 10 accessions each for zinc, iron, protein, calcium, and beta-carotene contents in finger millet, 5 accessions for high seed protein in chickpea, 14 accessions for zinc in pigeon pea seed [18]. Upadhyaya et al. [19] also carried out evaluation studies of core collection of foxtail millet and obtained protein content in the range of 10.7 to 18.5%, calcium from 90.3 to 288.7 mg/kg, iron from 24.1 to 68.0 mg/kg and zinc from 33.6 to 74.2 mg/kg.

### Diversity among core set accessions

A high level of variation was obtained for various mineral nutrients as revealed by the dendrogram. Results revealed that maximum variability among accessions was inter-specific and not limited to a particular country. The maximum diversity was observed among the accessions with origin from Syria and Turkey. The Euclidian distances calculated based on data from 0.845 to 14.291 showed large genetic distances among the accessions. Vieira et al. [29] reported genetic distances up to 196.61 in 19 wheat accessions analyzed for 17 phenotypic characters. A large genetic distance between heterotic germplasm can be useful for developing lines with good combining ability in hybrid breeding [30]. Earlier also, based on morphological and molecular studies, it has been established that Turkish lentil landraces had substantial genetic diversity at the genotypic and phenotypic levels [31]. Singh et al. [12] also reported maximum genetic diversity among wild *Lens* accessions with Turkey origin. Singh et al. [23] analyzed 50 lentil accessions for Fe and Zn content along with diversity analysis using 20 genomic and 54 EST-SSR markers which revealed role of geographic origin in diversity. The knowledge of the genetic diversity among accessions provides clues about the wider genetic base of accessions/species and that can be exploited through wide hybridization in order to develop polymorphic populations for various traits of interest.

### Principal Component Analysis

In present study of mineral composition of wild Lens accessions, PCA has been used to assess the pattern of variations among wild lentil accessions by considering all variables simultaneously where first four principal components accounted for 65.09% of the total variability. Similar findings were observed by Karakoy et al. [32] where first four PCs accounted for 79.45% of the variability. They reported PC1 accounted for 36.90% of the total variation, and P, Zn, Mg, and K had the highest positive coefficients, whereas, PC2 explained 20.38% of the total variation, and seed size, 100-seed weight, Mn, and Cu had the highest positive coefficients.

### Association studies between mineral elements and agro-morphological traits

The concentrations of several minerals, particularly Fe, Cu, Mn and Mo, were positively correlated with other minerals suggesting that similar pathways or transporters control the uptake and transport of these minerals. Karakoy et al. [32] reported similar observation for Zn in lentil samples. Cabrera et al. [33] also found a direct statistical correlation between Cu–Cr, Zn–Al and Cr–Ni and Al–Pb. The positive correlations among various minerals suggest the possibility of breeding for increased concentrations of these elements simultaneously. The variations in results from earlier studies can be explained on the basis that mineral composition is influenced by a number of factors, genetic background of the materials, and environmental adaptability [28].

## Conclusions and future perspectives

High variability was recorded for various essential minerals in wild lentil core accessions developed from global collection. Species *L*. *culinaris* ssp. *odemensis*, *L*. *culinaris* ssp. *orientalis* and *L*. *ervoides* showed remarkable variability for different minerals viz. Fe, Zn, Cu, Mn and Mo. In general, species, *L*. *nigricans* and *L*. *culinaris* ssp. *orientalis* showed wide variability for Ca content, whereas *L*. *ervoides* and *L*. *culinaris* ssp. *orientalis*, for Mg content. Accessions belonging to Turkey and Syria region showed maximum diversity for various minerals. The results highlight the underlying tight genetic control of physiological and molecular mechanisms involved in the mineral portioning. The genetic diversity analysis revealed intra- as well as inter-specific variability and relationships among the wild annual *Lens* core set accessions. Recently, some initiatives have been made for improvement of domesticated lentil through bio-fortification of minerals using cross breeding approach. But efficient pre-breeding efforts for utilizing wild *Lens* species for mineral bio-fortification of domesticated lentil improvement have not been undertaken at the global level. The present study clearly indicates the potential of wild lentil species through identification of essential mineral rich accessions including ILWL15, ILWL480 and ILWL401 etc. which can be used for bio-fortification of cultivated lentil using appropriate cross breeding techniques.

## Supporting information

S1 Table Elemental composition of core set accessions in comparison to the available literature values. (DOCX)

S2 Table Clustering pattern and Euclidean distances of core set accessions based on elemental composition data. (DOCX)

S3 Table Correlation among different minerals and between minerals and agro-morphological traits. (DOCX)
