# Supplementary material for: Bio-fortification potential of global wild annual lentil core collection
Source: PLoS One. 2018 Jan 18;13(1):e0191122. doi: 10.1371/journal.pone.0191122 (PMC5773171; doi:10.1371/journal.pone.0191122)
Supplement: S1 Table — (DOCX) [file pone.0191122.s001.docx]

**S1 Table. Elemental composition of core set accessions in comparison to the available literature values.**

| **Elements** | **Range obtained (mg/100 g)** | **Literature values (mg/100 g)** | **References** |
| --- | --- | --- | --- |
| **Na** | 30.0-318.0 | 10.85-23.00  30.60-79.00 | Laghetti et al. (2008)  Zia-Ul Haq et al. (2011) |
| **K** | 138.29-1578.00 | 916.8-1071.8  872-875  638-950  674.4-1061.2 | Laghetti et al. (2008)  Zia-Ul Haq et al. (2011)  Karakoy et al. (2012),  Alghamdi et al. (2014) |
| **P** | 37.50-593.75 | 405.4-542.4  292-294  286-533  286.9-546.7 | Laghetti et al. (2008)  Zia-Ul Haq et al. (2011)  Karakoy et al. (2012)  Alghamdi et al. (2014) |
| **Ca** | 4.74-188.75 | 49.57-59.79  118-121  48-128  64.9-84.8 | Laghetti et al. (2008)  Zia-Ul Haq et al. (2011)  Karakoy et al. (2012)  Alghamdi et al. (2014) |
| **Mg** | 15.0-159.00 | 85-126  126.1-157.3 | Karakoy et al. (2012)  Alghamdi et al. (2014) |
| **Fe** | 2.82-14.12 | 6.46-7.47  7.56-9.74  7.3 – 9.0  2.7-3.2  4.896-8.139  6.57-8.57  9.17-11.91 | Cabrera et al. (2003)  Laghetti et al. (2008)  Thavarajah et al. (2011)  Zia-Ul Haq et al. (2011)  Karakoy et al. (2012)  Alghamdi et al. (2014)  Leshe andTessema (2014) |
| **Zn** | 1.29-12.62 | 4.51-7.02  4.4-5.4  3.9-4.6  4.230-7.310  2.63-4.51  8.62-10.03 | Cabrera et al. (2003)  Thavarajah et al. (2011)  Zia-Ul Haq et al. (2011)  Karakoy et al. (2012)  Alghamdi et al. (2014)  Leshe andTessema (2014) |
| **Cu** | 0.50-7.12 | 0.20-0.33  0.92-1.61  8.9-9.9  0.910-1.692  0.86-1.37  0.226-0.282 | Cabrera et al. (2003)  Laghetti et al. (2008)  Zia-Ul Haq et al. (2011)  Karakoy et al. (2012)  Alghamdi et al. (2014)  Leshe andTessema (2014) |
| **Mn** | 1.22-9.99 | 12.07  1.4-4.3  1.150-1.620  1.26-2.85  6.7-8.2 | Kostova et al. (2008)  Zia-Ul Haq et al. (2011)  Karakoy et al. (2012)  Alghamdi et al. (2014)  Leshe andTessema (2014) |
| **Mo** | 1.02-11.89 | 1.56 | Kostova et al. (2008) |
| **Ni** | 0.16-3.50 | 0.01-0.033  0.120-0.244 | Cabrera et al. (2003)  Leshe andTessema (2014) |
| **Pb** | 0.01-0.58 | 0.04-0.06  0.142-0.176 | Cabrera et al. (2003)  Leshe andTessema (2014) |
| **Cd** | 0.00-0.03 | 0.0008-0.0010  0.009-0.013 | Cabrera et al. (2003)  Leshe andTessema (2014) |
| **Co** | 0.00-0.63 | 0.285-0.360 | Leshe andTessema (2014) |
