# Supplementary material for: Bio-fortification potential of global wild annual lentil core collection
Source: PLoS One. 2018 Jan 18;13(1):e0191122. doi: 10.1371/journal.pone.0191122 (PMC5773171; doi:10.1371/journal.pone.0191122)
Supplement: S2 Table — (DOCX) [file pone.0191122.s002.docx]

**S2 Table. Clustering pattern and Euclidean distances of core set accessions based on elemental composition data.**

| **Number of Clusters** | **Distance** |  | **Leader** | **Origin** | **Joiner** | **Origin** |
| --- | --- | --- | --- | --- | --- | --- |
| 95 | 0.84515203 |  | ILWL 95 | Turkey | ILWL 35 | Turkey |
| 94 | 0.90088965 |  | ILWL 19 | Spain | EC718270 | Croatia |
| 93 | 1.23879366 |  | ILWL 75 | Israel | ILWL 198 | Syria |
| 92 | 1.24309362 |  | ILWL 195 | Syria | EC718673 | Syria |
| 91 | 1.33381030 |  | ILWL 235 | Syria | ILWL 66(B) | Turkey |
| 90 | 1.35899608 |  | ILWL 8 | Turkey | ILWL 65 | Turkey |
| 89 | 1.40050740 |  | ILL 8006 | Syria | ILL 10829 | Syria |
| 88 | 1.40731741 |  | ILWL 63 | Turkey | ILWL 234 | Syria |
| 87 | 1.50035889 |  | ILWL 292 | Turkey | ILWL 414 | Syria |
| 86 | 1.53963583 |  | ILWL 31 | Spain | ILWL 38 | Turkey |
| 85 | 1.54849206 |  | ILWL 166 | Syria | ILWL 436 | Turkey |
| 84 | 1.55213012 |  | ILWL 14 | Syria | ILWL 19 | Spain |
| 83 | 1.59513176 |  | ILWL 276 | Turkey | ILWL 321 | Turkey |
| 82 | 1.61218580 |  | ILWL 117 | Syria | ILWL 480 | Syria |
| 81 | 1.62461471 |  | ILWL 7 | Turkey | ILWL 95 | Turkey |
| 80 | 1.66612160 |  | EC718273 | Spain | ILWL 50 | Croatia |
| 79 | 1.68284163 |  | ILWL 75 | Israel | ILWL 181 | Syria |
| 78 | 1.71560151 |  | ILWL 8 | Turkey | ILWL 61 | Turkey |
| 77 | 1.72526344 |  | ILWL 89 | Turkey | ILWL 344 | Syria |
| 76 | 1.73187479 |  | ILWL 14 | Syria | ILWL 18 | France |
| 75 | 1.74833217 |  | ILWL 292 | Turkey | ILWL 408 | Syria |
| 74 | 1.78116640 |  | ILWL 15 | France | ILWL 22 | Italy |
| 73 | 1.82394495 |  | ILWL 195 | Syria | ILWL 199 | Syria |
| 72 | 1.84530689 |  | ILWL 101 | Turkey | ILWL 349 | Syria |
| 71 | 1.86114843 |  | ILWL 29 | Spain | ILWL 56 | Palestine |
| 70 | 1.86919278 |  | ILWL 90 | Turkey | ILWL 235 | Syria |
| 69 | 1.87888686 |  | ILWL 124 | Syria | ILWL 14 | France |
| 68 | 1.90823391 |  | ILWL 243 | Syria | ILWL 357 | Syria |
| 67 | 1.92220611 |  | ILWL 58 | Turkey | ILWL 401 | Lebanon |
| 66 | 1.95352979 |  | ILWL 37 | Turkey | ILWL 165 | Syria |
| 65 | 2.00816388 |  | EC718275 | Turkey | EC718692 | France |
| 64 | 2.01136401 |  | ILWL 230 | Syria | ILWL 246 | Syria |
| 63 | 2.02407470 |  | ILWL 196 | Not Known | EC718694 | Syria |
| 62 | 2.03447064 |  | ILWL 37 | Turkey | ILWL 166 | Syria |
| 61 | 2.05442120 |  | ILWL 361 | Syria | ILWL 409 | Syria |
| 60 | 2.05457704 |  | EC718266 | Italy | ILWL 167 | Syria |
| 59 | 2.07541881 |  | ILWL 34 | Ukraine | ILWL 15 | France |
| 58 | 2.08283040 |  | ILWL 14 | Syria | ILWL 460 | Turkey |
| 57 | 2.14304402 |  | ILWL 16 | Alpes-Cote d’ Azur | ILWL 308 | Turkey |
| 56 | 2.19064414 |  | ILWL 191 | Croatia | EC718273 | Spain |
| 55 | 2.20475140 |  | ILWL 330 | Syria | ILWL 443 | Turkey |
| 54 | 2.21694891 |  | ILWL 51 | Montenegro | ILWL 292 | Turkey |
| 53 | 2.23271238 |  | ILWL 476 | Turkey | ILWL 269 | Turkey |
| 52 | 2.28243593 |  | ILWL 243 | Syria | ILWL 361 | Syria |
| 51 | 2.29469215 |  | ILWL 442 | Turkey | EC718439 | Israel |
| 50 | 2.30626497 |  | ILWL 15 | France | ILWL 305 | Turkey |
| 49 | 2.34730971 |  | ILWL 243 | Syria | ILWL 438 | Turkey |
| 48 | 2.42795668 |  | ILWL 92 | Turkey | ILWL 276 | Turkey |
| 47 | 2.43580316 |  | ILWL 8 | Turkey | ILWL 75 | Israel |
| 46 | 2.44753060 |  | ILWL 7 | Turkey | ILWL 20 | Palestine |
| 45 | 2.46724010 |  | ILWL 30 | Spain | ILWL 63 | Turkey |
| 44 | 2.47268431 |  | ILWL 90 | Turkey | ILWL 60 | Turkey |
| 43 | 2.50673941 |  | ILWL 43 | Croatia | ILWL 441 | Turkey |
| 42 | 2.51694139 |  | ILWL 89 | Turkey | ILWL 96 | Turkey |
| 41 | 2.52429703 |  | ILWL 51 | Montenegro | ILWL 418 | Syria |
| 40 | 2.53462007 |  | ILWL 117 | Syria | EC718275 | Turkey |
| 39 | 2.64737672 |  | ILWL 243 | Syria | ILWL 320 | Turkey |
| 38 | 2.70397349 |  | ILWL 196 | Not Known | ILWL 58 | Turkey |
| 37 | 2.71664640 |  | ILWL 476 | Turkey | ILWL 480 | Syria |
| 36 | 2.77935827 |  | ILWL 124 | Syria | ILWL 343 | Syria |
| 35 | 2.97896326 |  | ILWL 101 | Turkey | ILWL 230 | Syria |
| 34 | 3.00605088 |  | ILWL 117 | Syria | ILWL 196 | Not Known |
| 33 | 3.03391234 |  | ILWL 29 | Spain | EC718311 | Israel |
| 32 | 3.07276220 |  | ILWL 15 | France | ILWL 31 | Spain |
| 31 | 3.08285750 |  | ILWL 7 | Turkey | ILWL 195 | Syria |
| 30 | 3.21439486 |  | ILWL 43 | Croatia | ILWL 92 | Turkey |
| 29 | 3.24947119 |  | ILWL 398(A) | Lebanan | ILWL 442 | Turkey |
| 28 | 3.33150641 |  | ILWL 16 | Alpes-Cote d’ Azur | ILWL 191 | Croatia |
| 27 | 3.42872124 |  | ILWL 89 | Turkey | ILWL 51 | Montenegro |
| 26 | 3.47290961 |  | ILWL 16 | Alpes-Cote d’ Azur | ILWL 34 | Ukraine |
| 25 | 3.54125207 |  | ILWL 14 | Syria | ILWL 15 | France |
| 24 | 3.61675966 |  | ILWL 227 | Syria | ILWL 398(A) | Lebanan |
| 23 | 3.62414259 |  | ILWL 243 | Syria | ILWL 429 | Spain |
| 22 | 3.70370837 |  | ILWL 124 | Syria | ILWL 9 | Syria |
| 21 | 3.77684903 |  | ILWL 90 | Turkey | ILWL 30 | Spain |
| 20 | 3.82220716 |  | ILWL 16 | Alpes-Cote d’ Azur | EC718266 | Italy |
| 19 | 3.97766285 |  | ILWL 278 | Turkey | ILWL 476 | Turkey |
| 18 | 4.03469732 |  | ILWL 8 | Turkey | ILWL 89 | Turkey |
| 17 | 4.03943236 |  | ILWL 7 | Turkey | ILWL 37 | Turkey |
| 16 | 4.62201780 |  | ILWL 278 | Turkey | ILWL 330 | Syria |
| 15 | 4.64313400 |  | ILWL 101 | Turkey | ILWL 14 | Syria |
| 14 | 4.79779606 |  | ILWL 227 | Syria | ILL 8006 | Syria |
| 13 | 5.03305931 |  | ILWL 16 | Alpes-Cote d’ Azur | ILWL 29 | Spain |
| 12 | 5.23566925 |  | ILWL 359 | Syria | ILWL 90 | Turkey |
| 11 | 5.45792172 |  | ILWL 7 | Turkey | ILWL 124 | Syria |
| 10 | 6.23629938 |  | ILWL 8 | Turkey | ILWL 359 | Syria |
| 9 | 6.60839904 |  | ILWL 278 | Turkey | ILWL 384 | Tajikistan |
| 8 | 6.88616283 |  | ILWL 117 | Syria | ILWL 243 | Syria |
| 7 | 6.94590421 |  | ILWL 227 | Syria | ILWL 43 | Croatia |
| 6 | 7.39232274 |  | ILWL 101 | Turkey | ILWL 117 | Syria |
| 5 | 7.74757628 |  | ILWL 7 | Turkey | ILWL 8 | Turkey |
| 4 | 8.26858839 |  | ILWL 278 | Turkey | ILWL 16 | Alpes-Cote d’ Azur |
| 3 | 10.51837221 |  | ILWL 7 | Turkey | ILWL 101 | Turkey |
| 2 | 11.24858930 |  | ILWL 227 | Syria | ILWL 278 | Turkey |
| 1 | 14.29077413 |  | ILWL 7 | Turkey | ILWL 227 | Syria |
