# Supplementary material for: Bio-fortification potential of global wild annual lentil core collection
Source: PLoS One. 2018 Jan 18;13(1):e0191122. doi: 10.1371/journal.pone.0191122 (PMC5773171; doi:10.1371/journal.pone.0191122)
Supplement: S3 Table — (DOCX) [file pone.0191122.s003.docx]

**S3 Table. Correlation among different minerals and between minerals and agro-morphological traits.**

|  | Na | K | P | Ca | Mg | Fe | Zn | Cu | Mn | Mo | Ni | Pb | Cd | Co | As |
| --- | --- | --- | --- | --- | --- | --- | --- | --- | --- | --- | --- | --- | --- | --- | --- |
| Na | 1.00 |  |  |  |  |  |  |  |  |  |  |  |  |  |  |
| K | .21(*) | 1.00 |  |  |  |  |  |  |  |  |  |  |  |  |  |
| P | -.31(**) | -0.12 | 1.00 |  |  |  |  |  |  |  |  |  |  |  |  |
| Ca | .18(*) | -0.08 | -.34(**) | 1.00 |  |  |  |  |  |  |  |  |  |  |  |
| Mg | 0.72(**) | .21(*) | -.31(**) | .18(*) | 1.00 |  |  |  |  |  |  |  |  |  |  |
| Fe | 0.01 | .24(**) | -0.01 | -.21(*) | 0.01 | 1.00 |  |  |  |  |  |  |  |  |  |
| Zn | -0.01 | .26(**) | 0.05 | -0.16 | -0.01 | .79(**) | 1.00 |  |  |  |  |  |  |  |  |
| Cu | -0.08 | -0.10 | .30(**) | -0.08 | -0.08 | 0.10 | .18(*) | 1.00 |  |  |  |  |  |  |  |
| Mn | -0.03 | -0.09 | 0.13 | 0.10 | -0.03 | -0.09 | 0.02 | .75(**) | 1.00 |  |  |  |  |  |  |
| Mo | 0.06 | 0.16 | -.30(**) | .22(*) | 0.06 | 0.08 | 0.10 | .33(**) | .44(**) | 1.00 |  |  |  |  |  |
| Ni | 0.11 | 0.11 | -0.04 | 0.12 | 0.11 | -0.02 | 0.02 | .29(**) | .44(**) | .24(**) | 1.00 |  |  |  |  |
| Pb | -0.06 | .23(*) | -.17(*) | .22(*) | -0.06 | .17(*) | .24(**) | .25(**) | .28(**) | .43(**) | 0.12 | 1.00 |  |  |  |
| Cd | -0.01 | 0.01 | -0.10 | .33(**) | -0.01 | -0.01 | 0.05 | .48(**) | .51(**) | .61(**) | 0.16 | .52(**) | 1.00 |  |  |
| Co | -0.03 | -.28(**) | -0.06 | .22(*) | -0.03 | -.29(**) | -.24(**) | .24(**) | .58(**) | 0.17 | .43(**) | 0.07 | .23(*) | 1.00 |  |
| As | 0.09 | 0.10 | -0.16 | -0.02 | 0.09 | .18(*) | 0.12 | .26(**) | .25(**) | .25(**) | .21(*) | 0.13 | .22(*) | 0.15 | 1.00 |
| DF | .28(**) | .33(**) | -.19(*) | -0.14 | .28(**) | .22(*) | .17(*) | -0.05 | -.17(*) | 0.08 | -0.17 | -0.02 | -0.04 | -.39(**) | 0.08 |
| DM | .27(**) | .29(**) | -.22(*) | -0.04 | .27(**) | .22(*) | .20(*) | -0.05 | -0.17 | 0.12 | -0.13 | 0.03 | 0.01 | -.36(**) | 0.08 |
| PH | 0.09 | -0.01 | -0.11 | 0.17 | 0.09 | -0.02 | -0.01 | 0.06 | -0.09 | -0.08 | 0.01 | 0.06 | 0.04 | -.20(*) | 0.02 |
| NB | 0.05 | -0.07 | 0.01 | -0.10 | 0.05 | 0.13 | 0.09 | 0.00 | 0.12 | 0.06 | 0.02 | 0.00 | -0.12 | .17(*) | 0.03 |
| NPP | 0.02 | -0.07 | .21(*) | 0.00 | 0.02 | 0.02 | -0.02 | -0.03 | 0.05 | -0.12 | -0.02 | -0.02 | -0.13 | 0.07 | -0.09 |
| NSPL | 0.04 | -0.06 | .19(*) | -0.02 | 0.04 | 0.04 | 0.00 | -0.03 | 0.05 | -0.11 | -0.03 | -0.02 | -0.14 | 0.05 | -0.08 |
| NSPD | -0.05 | -0.04 | -0.15 | -.21(*) | -0.05 | 0.15 | 0.08 | -0.14 | -.18(*) | -0.01 | -.19(*) | -0.02 | -0.08 | -0.17 | 0.17 |
| SW | -.21(*) | 0.11 | -0.07 | 0.04 | -.21(*) | -0.01 | 0.00 | -0.08 | -0.01 | 0.16 | 0.15 | .18(*) | 0.05 | 0.10 | 0.10 |
| SY | -0.03 | -0.02 | 0.06 | 0.02 | -0.03 | 0.01 | -0.01 | -0.02 | 0.05 | 0.01 | -0.01 | 0.09 | -0.06 | 0.07 | -0.02 |
| BYd | -0.03 | -0.11 | 0.14 | -0.03 | -0.03 | 0.01 | 0.00 | 0.12 | 0.15 | -0.01 | 0.07 | 0.09 | -0.05 | .24(**) | 0.02 |
| RR | -0.02 | 0.15 | -.22(*) | 0.06 | -0.02 | -0.02 | -0.07 | -0.16 | -0.16 | 0.06 | -0.13 | -0.15 | 0.02 | -.21(*) | 0.02 |
| RPM | 0.15 | .21(*) | -.28(**) | 0.16 | 0.15 | 0.04 | -0.05 | -0.06 | 0.00 | .19(*) | -0.12 | -0.03 | 0.05 | -0.15 | 0.11 |
| LPA | 0.00 | 0.00 | 0.07 | -0.01 | 0.00 | 0.01 | 0.12 | .18(*) | .20(*) | -0.09 | .24(**) | -0.13 | -0.07 | 0.00 | 0.04 |
| LPS | -0.12 | -.22(*) | .22(*) | -0.04 | -0.12 | 0.03 | -0.05 | .18(*) | 0.06 | 0.07 | -0.08 | 0.08 | 0.13 | 0.07 | -0.06 |
| LPD | 0.15 | .25(**) | -.28(**) | 0.04 | 0.15 | -0.02 | -0.04 | -.37(**) | -.27(**) | 0.00 | -0.12 | 0.00 | -0.13 | -0.10 | 0.01 |
| LLSS | -0.01 | -.26(**) | 0.16 | .26(**) | -0.01 | -0.15 | -0.08 | 0.09 | 0.14 | -0.04 | -0.04 | -0.06 | .22(*) | .23(*) | -0.14 |
| LLSM | 0.11 | .33(**) | -.23(*) | -.23(*) | 0.11 | .21(*) | 0.14 | -0.06 | -.17(*) | 0.11 | 0.07 | 0.02 | -0.13 | -.20(*) | 0.16 |
| LLSL | -0.16 | -0.11 | 0.12 | -0.05 | -0.16 | -0.10 | -0.10 | -0.06 | 0.05 | -0.12 | -0.06 | 0.06 | -0.16 | -0.05 | -0.04 |
| TLR | -0.09 | 0.02 | 0.04 | 0.05 | -0.09 | -0.03 | 0.07 | 0.17 | .22(*) | .24(**) | 0.16 | 0.14 | .18(*) | .19(*) | 0.08 |
| TLP | 0.09 | -0.02 | -0.04 | -0.05 | 0.09 | 0.03 | -0.07 | -0.17 | -.22(*) | -.24(**) | -0.16 | -0.14 | -.18(*) | -.19(*) | -0.08 |
| PSN | -.39(**) | -0.09 | .30(**) | -0.16 | -.39(**) | -0.01 | -0.05 | -0.03 | -0.10 | -.24(*) | 0.00 | -0.03 | -0.16 | -0.02 | -0.09 |
| PSL | 0.15 | 0.04 | 0.11 | .17(*) | 0.15 | 0.12 | 0.12 | .21(*) | .20(*) | 0.13 | .26(**) | 0.11 | .18(*) | 0.03 | .18(*) |
| PSM | 0.05 | 0.14 | -0.15 | -0.15 | 0.05 | 0.03 | -0.04 | -0.05 | -0.02 | 0.01 | -0.09 | 0.04 | 0.06 | 0.09 | 0.04 |
| PSH | -0.05 | -0.15 | -0.01 | 0.07 | -0.05 | -0.12 | -0.03 | -0.09 | -0.10 | -0.05 | -0.10 | -0.11 | -0.14 | -0.11 | -0.15 |
| PDN | -.39(**) | -0.09 | .29(**) | -0.16 | -.39(**) | -0.01 | -0.05 | -0.03 | -0.10 | -.24(*) | 0.00 | -0.03 | -0.16 | -0.02 | -0.09 |
| PDL | 0.07 | -0.04 | 0.02 | .26(**) | 0.07 | -0.02 | 0.12 | 0.14 | 0.11 | 0.01 | .24(**) | 0.04 | 0.09 | 0.03 | 0.07 |
| PDM | 0.14 | .20(*) | -.22(*) | -0.16 | 0.14 | 0.11 | 0.04 | -.21(*) | -.28(**) | 0.06 | -.39(**) | -0.08 | -0.08 | -.28(**) | 0.03 |
| PDH | -0.08 | -0.16 | 0.13 | 0.02 | -0.08 | -0.11 | -0.13 | 0.14 | .25(**) | 0.01 | .25(**) | 0.06 | 0.08 | .29(**) | -0.05 |

*Correlation is significant at the 0.05 level, **Correlation is significant at the 0.01 level.

DF-days to flowering, DM-days to maturity, PH-plant height, NB-number of branches, NPP-number of pods per plant, NSPL-number of seeds per plant, NSPD-number of seeds per pod, SW-seed weight, SY-seed yield, BYD-biological yield, RR-rust resistance, RPM-resistance to powdery mildew, LPA-leaf pubescence absent, LPS-leaf pubescence slight, LPD-leaf pubescence dense, LLSS-leaflet size small, LLSM-leaflet size medium, LLSL-leaflet size large, TLR-tendril length rudimentary, TLP-tendril length prominent, PSN-pod shedding none, PSL-pod shedding low, PSM-pod shedding medium, PSH-pod shedding high, PDN-pod dehiscence none, PDL-pod dehiscence low, PDM-pod dehiscence medium, PDH-pod dehiscence high
